# Reverse shoulder arthroplasty leads to significant biomechanical changes in the remaining rotator cuff

**DOI:** 10.1186/1749-799X-6-42

**Published:** 2011-08-16

**Authors:** Sebastian Herrmann, Christian König, Markus Heller, Carsten Perka, Stefan Greiner

**Affiliations:** 1 Center for Musculosceletal Surgery, Charité-Universitätsmedizin Berlin, Charitéplatz 1, D-10117 Berlin, Germany; 2 Julius Wolff Institute, Charité - Universitätsmedizin Berlin, Center for Sports Science and Sports Medicine Berlin (CSSB) Philippstr. 13, 10115 Berlin, Germany

**Keywords:** shoulder arthroplasty, cuff tear arthropathy, reverse shoulder prosthesis, biomechanics shoulder, moment arms, rotator cuff

## Abstract

**Objective:**

After reverse shoulder arthroplasty (RSA) external and internal rotation will often remain restricted. A postoperative alteration of the biomechanics in the remaining cuff is discussed as a contributing factor to these functional deficits.

**Methods:**

In this study, muscle moment arms as well as origin-to-insertion distance (OID) were calculated using three-dimensional models of the shoulder derived from CT scans of seven cadaveric specimens.

**Results:**

Moment arms for humeral rotation are significantly smaller for the cranial segments of SSC and all segments of TMIN in abduction angles of 30 degrees and above (p ≤ 0.05). Abduction moment arms were significantly decreased for all segments (p ≤ 0.002). OID was significantly smaller for all muscles at the 15 degree position (p ≤ 0.005), apart from the cranial SSC segment.

**Conclusions:**

Reduced rotational moment arms in conjunction with the decrease of OID may be a possible explanation for the clinically observed impaired external and internal rotation.

## Background

Promising early functional results can be achieved with reverse shoulder arthroplasty (RSA), especially in patients with severe cuff tear arthropathy[1-3]. It also is a salvage procedure for fracture sequelae[4-7] and revision of failed hemiarthroplasty[8,9], even though outcome is less predictable in these patients.

Patients suffering from the above conditions experience severe restrictions in their activities of daily living by either loss of function due to the insufficient rotator cuff or pain. Even though functional impairment can be extensive and all parts of the cuff can be affected, M. supra-and M. infraspinatus seem to be the most commonly involved, whereas teres minor and subscapularis often remains intact [10].

One mechanism by which RSA improves function is the increase of the deltoids moment arm by shifting the centre of rotation medially. Additionally the deltoid's proportion, contributing to active elevation, is enlarged and the hemispheric design provides stability and constraint. These changes result in a significantly improved ability to actively abduct and forward-flex the arm[11], while internal and external rotation often remains impaired or even decreases postoperatively[12].

Previous studies have given a thorough insight into the biomechanics of the shoulder joint after RSA including joint forces and deltoid function[13], transfer procedures[14] and strategies to avoid inferior impingement[15,16]. However, so far it remains unclear why functional deficits in internal/external rotation can occur, even though the muscles mainly responsible for this function remain intact.

We hypothesised that RSA reduces the moment arms and the origin-to-insertion distance (OID) of subscapularis (SSC) and teres minor (TMI), which in healthy shoulders are responsible for internal/external rotation.

The aim of this study was therefore to analyse how RSA changes the moment arms and the OID of the SSC and TMI during glenohumeral abduction before and after RSA using a combined in-vitro/in-silico approach, where in silico refers to a virtual, computational model.

## Methods

### Specimens

Shoulder specimens of seven fresh frozen human specimens (mean age 77 years, range 63-84 years) were tested. All donors have consented participation in the institutional body donor program, which is approved by local authorities. None of these shoulders showed signs of previous surgery, trauma, deformities or distinct osteoarthritis. There were five right and two left shoulders. Image data of the left specimens were mirrored with respect to the sagittal plane, so definitions of right shoulder were applicable.

### Specimen preparation

After thawing, careful dissection of all specimens was undertaken. Excessive soft tissue was removed so muscle origins and insertions of subscapularis and teres minor could be visualised. To mark the bony insertion sites of both muscles their outermost limits were marked with radio opaque markers. Bony landmarks including the medial and lateral epicondyle, angulus acromialis, trigonum scapulae and angulus inferior were also marked with markers to ensure an accurate repetitive landmark acquisition.

Thin sliced computed tomography (Aquilion 64, Toshiba Medical Systems) with a resolution of 512 × 512 and a slice thickness of 0.5 mm was performed.

Using a 3D data visualization, analysis and modelling software (AMIRA; Mercury Computer Systems, Chelmsford, MA, USA), the spatial position of all previously marked landmarks was determined and 3D models of the humerus and the scapula were created for each specimen.

Thereafter a polycarbonate resin model of a reverse prosthesis (Mathys AG, Bettlach, Switzerland) was implanted by an experienced orthopaedic surgeon following the standard surgical protocol. The humeral component was implanted in ten degrees of retroversion as measured by the forearm axis, according to our clinical practice to avoid anterior or posterior impingement. The glenoid component was implanted so a slight inferior overhang could be observed. Height of the humeral component was adjusted so substantial deltoid tension and therefore sufficient joint stability was gained. The prosthesis resembles the company's reverse prosthesis model (Affinis Inverse^®^). The advantage of the polycarbonate material was the prevention of radiologic artefacts, which allowed reconstruction of the proximal humerus anatomy with high accuracy. The same implant size was used for all specimens (glenoid component 39 mm, humeral component stem 6/110 mm).

After the implantation the CT scans were repeated and the position of the prostheses components relative to the bones determined in each specimen.

### Definition of the joint coordinate systems

**Figure 1:**
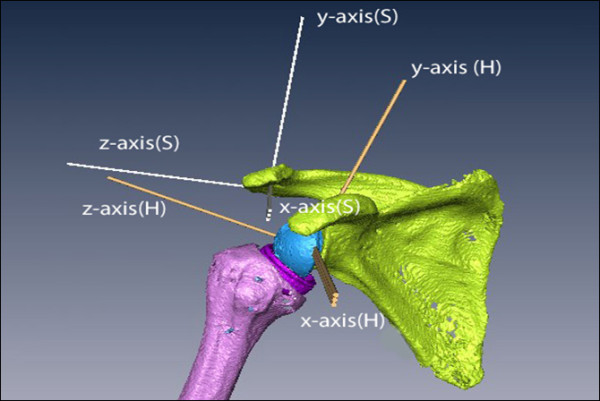
**Three-Dimensional shoulder model created from CT-scans after implantation of a polycarbonate-resin inverse shoulder prosthesis**. Two coordinate systems (Scapula (S); Humerus (H)) were defined according to the recommendations of the Society of Biomechanics.

In the 3D surface models of each specimen joint coordinate systems (CS) were defined in the scapula and the humerus according to the recommendations of the International Society of Biomechanics[17] (Figure 1). In brief, the scapula CS originates at the angulus acromialis and is defined by three bony, scapular landmarks. The coordinate system's x-axis is pointing anteriorly; the y-axis cranially and the z-axis laterally. The humeral CS is defined by two bony landmarks, the medial and the lateral epicondyle and the centre of the humeral head. The anatomical direction of the axes was equivalent to the scapula CS. To determine the centre of the humeral head a sphere was fitted into the computer model of the humeral articular surface, using a least-square fit algorithm[18]. In the postoperative condition after RSA, the centre of the articular surface of the glenoid component was determined to define the centre of rotation in postoperative shoulders. For easy and distinct interpretation in line with clinical practice the following definition for functional moments was used: a positive moment arm in regard to the scapular z-axis, describes the potential of anteflexion. Respectively negative values describe the muscles potential of retroversion. The humeral y-axis is the rotational axis. A positive moment arm around this axis stands for capability to internally rotate the humerus, negative values result in potential external rotation. Finally the x-axis of the scapular coordinate system is considered the axis for abduction and adduction. Positive values indicating abduction capability; negative values adduction potential.

Humeral position was expressed in the scapula CS.

### Analysis of moment arms

All moment arms and the origin-to-insertion distance were calculated in the three-dimensional, virtual model derived from the CT scans.

Since the relative position of the humerus to the scapula could not be accurately set during the CT scan, the rotations were calculated that transformed the humerus to the scapula CS, defining a zero degree position. To analyse a representative range of motion (ROM) in glenohumeral abduction, conditions of 15, 30, 45 and 60° abduction were simulated by virtually rotating the humerus around the humeral anterior-posterior (x) axis.

**Figure 2:**
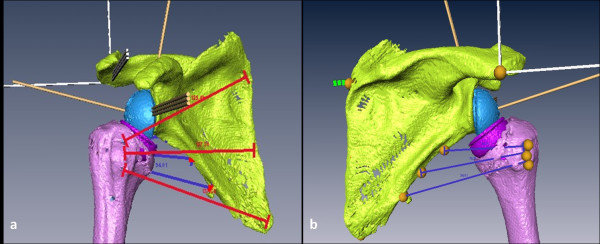
**Three Muscle-Segments were defined by virtual lines from its origin to its insertion for a: M.subscapularis and b: M.teres minor**.

To calculate moment arms for M. subscapularis and M. teres minor the radio opaque markers which were placed in the specimen were identified in the CT scan and the muscles modelled as lines between the muscles' origin and insertion. Since the markers only represented the outermost boundaries of the muscles, a third line was defined in the middle of these two lines (Figure 2). Wrapping of these muscles was not considered.

Moment arms for abduction/adduction, anteflexion/retroversion and external/internal rotation for these three segments of each muscle were acquired using the origin-to-insertion method which is described elsewhere in more detail[19]. In brief, to calculate the moments for the individual rotations, the total moment is multiplied with the unit vector pointing in the direction of the axis of that specific rotation.

$$M_{rot\text{\_}axis}=\left(\vec{r}\times \vec{F}\right)∙{\vec{e}}_{rot\text{\_}axis}$$

The moment arms for each rotation (*l*_*rot*_*axis*_) were then obtained by dividing the calculated moment by the absolute value of the acting force.

$$l_{rot\text{\_}axis}=\frac{\left(\vec{r}\times \vec{F}\right)}{\left|\vec{F}\right|}∙{\vec{e}}_{rot\text{\_}axis}$$

$$l_{rot}^{hum}=\left({\vec{r}}^{hum}\times {\vec{u}}^{hum}\right)∙{\vec{e}}_{y}^{hum}=r_{z}^{hum}u_{x}^{hum}-r_{x}^{hum}u_{z}^{hum}$$

Simplification of the formula allowed using the unit vector of the acting force $\vec{u}$ instead of specific muscle forces. The moment arms are therefore dependent on the vector ($\vec{r}$) pointing from the centre of rotation to the point of muscle force application and the direction of the force ($\vec{u}$). Moment arms for external/internal rotation were calculated with respect to the y-axis of the humerus coordinate system, while the abduction/adduction and anteflexion/retroversion moment arms were calculated with respect to the x- and the z-axis of the scapula co ordinate system respectively. These calculations were repeated for each abduction position.

To estimate how the muscle tension may be influenced by RSA, the length of the previously defined muscle lines was determined pre- and postoperatively. A shorter distance postoperatively is indicative of a decreased muscle tension.

Pre- and postoperative moment arms as well as origin-to-insertion distance for subscapularis and teres minor were analysed for statistical differences using the independent, two-sided Student's t-test.

## Results

### Subscapularis

**Figure 3:**
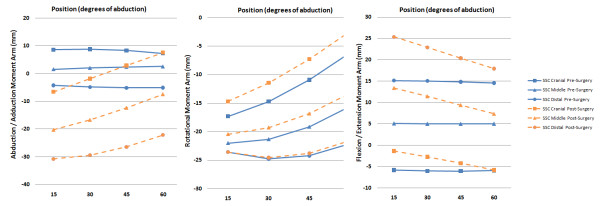
**Moment Arms for Abduction/Adduction, Rotation and Flexion/Extension for all three segments of subscapularis before and after RSA**.

There was a significant change of abduction moment arm values for all three muscle segments in all tested positions after reverse arthroplasty (p ≤ 0.0012), except for the most cranial segment at 60 degree abduction (p = 0.86)(Figure 3). In the pre-operative group, the calculated moment arms resulted in small abduction capacity as observed in the cranial segments, to small adductive moment arms in the distal segments. In postoperative shoulders all segments had significant bigger adduction moment arms (p ≤ 0.05), indicating an increased potential in generating adductive forces, whereas the abduction-potential will be lost.

Postoperative rotational moment arms of the two more cranial segments were significantly smaller at all positions (p ≤ 0.05), whereas no difference could be seen for the distal segment (p ≥ 0.45).

**Figure 4:**
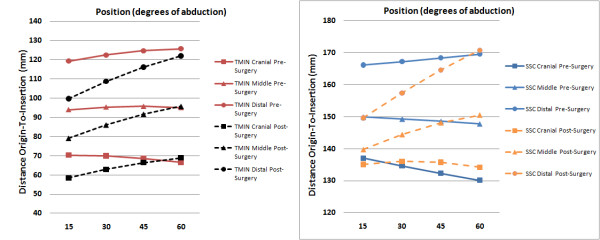
**Origin-to-Insertion distance for all segments of subscapularis and teres minor before and after RSA**.

Origin-to-insertion distances of the two distal segments decreased significantly after RSA at the 15 degree position (p ≤ 0.005) and of most distal segment only at the 30 degree position (p = 0.003). No difference in length was seen for the other positions (Figure 4).

### Teres minor

**Figure 5:**
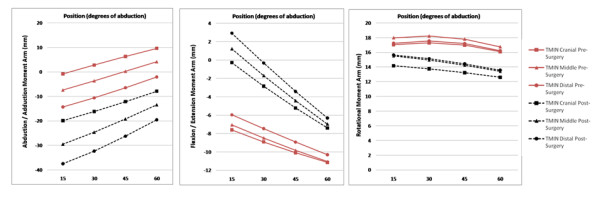
**Moment Arms for Abduction/Adduction, Rotation and Flexion/Extension for all three segments of teres minor before and after RSA**.

In the postoperative group, significantly bigger negative values for abduction/adduction (x-axis) moment arm could be seen (p ≤ 0.0005), indicating a higher potential of generating an adduction force (Figure 5). Contrary to the postoperative group, positive values for one or two cranial segments could be seen at the 45 and 60 degree position in pre-operative shoulders indicating an abductive potential of these segments.

While no difference was seen for rotational moment arms at the 15 degree position, values were significantly smaller with increasing abduction angle in the postoperative group (p ≤ 0.05).

Small negative values for flexion/extension moment arm could be seen, with no statistical differences between the two groups.

Origin to insertion distance was significantly smaller for all segments at 15 and 30 degrees abduction (p ≤ 0.005). The overall differences ranged from 7 to 20 mm. At 45 degrees this differences could only be observed for the two distal segments. At 60 degrees abduction no difference in muscle length was determined (Figure 4).

## Discussion

This study aimed to analyse moment arms and origin-to-insertion distance of the subscapularis and teres minor before and after reverse shoulder arthroplasty using a combined in-vitro/in-silico approach. Even though the functionally deficient infraspinatus may contribute to a loss of external rotation, the aim of this study was to investigate the effect RSA has on the intact muscles and their capability to perform rotational movements. Therefore the function of the infraspinatus was not specifically analysed in this study. This is the first study to characterise these properties after RSA. Knowledge of the functional properties of these muscles is of enormous importance for clinical practice and possible further improvement on prosthesis design or surgical technique.

Our pre-operative group consists of healthy shoulders, in which the humeral head is centered in the glenoid cavity. This might not be the case in shoulders with cuff tear arthropathy, but as the position of the humerus and therefore the center of rotation is highly variable in this pathology, we assumed this not practicable in terms of reproducibility. However, we assume in cases with a significantly cranialised humeral head the overall distalisation will be even more pronounced, leading to even more substantial changes in the joint's biomechanics.

The humeral component was implanted in ten degrees of retroversion in our entire specimens. Varying the humeral components' rotational alignment will likely have an impact on muscle tension. However in our opinion it is not an option to decrease muscle slackening as, for example, tensioning the posterior cuff will result in reduced tension of the anterior segments and vice versa. Also an increased retroversion might result in increased prosthetic impingent in neutral rotation or even increase the risk of prosthetic dislocation.

The methodology used is based on three-dimensional models derived from CT specimens' data. While CT scans allow reconstruction of the osseous anatomy with high precision, accuracy for identification of muscle origins and insertions was assumed not to be high enough. Therefore we marked muscle origins and insertions after preparation and visualisation using radio-opaque markers. Muscle wrapping was not included in this model, as it was considered negligible in the tested positions. Nonetheless we are aware of its possible impact to the overall value of our results. However, in our study we aimed to analyse the change of muscle properties rather than to obtain absolute values. The possible inaccuracy was therefore assumed acceptable. The pre-operative moment arm values calculated using this method are comparable to data from previous studies concerning normal shoulders [20,21].

One of the major drawbacks of RSA is its lacking potential to improve active external and internal rotation. While in healthy shoulders external rotation is dependent on teres minor integrity, after RSA potential of external rotation remains small irrespective of its pre-operative status. Even in patients with only mild fatty degeneration preoperatively, the gain in active external rotation remains small. Patients with higher grade fatty infiltration pre-operatively, might even experience a loss in external rotation[22]. While Boileau et al. [23] propose several reasons, such as prosthesis design and altered biomechanical properties of the deltoid, as being responsible for this, postoperative changes to the teres minor's rotational moment arms and origin-to-insertion distance, as shown in our study might be another, important contributing factor. Rotational moment arms are significantly smaller for all but the 15 degrees position, even though a corresponding trend in this position can be seen as well. Additionally muscle slackening might further reduce its efficiency, as origin-to-insertion distance is significantly smaller, especially in the 15 degrees position, reaching up to 20 mm for the distal segment.

Accordingly internal rotation, which in healthy shoulders depends on intact subscapularis function, often is compromised after RSA as well[24]. The subscapularis muscle tendon unit is the main internal rotator and contributes considerably to active stabilisation of the glenohumeral joint. In this study the two more cranial segments had significant smaller rotational moment arms after RSA, while no difference could be seen for the distal segment. No definite rational can be given to explain this difference. Further mathematical analysis might therefore be necessary.

While failed or non-performed reconstruction of the subscapularis has shown to have an influence on clinical outcome[25] in anatomical shoulder arthroplasty, no difference was seen after RSA at this stage[26]. Even though Edwards et al. [27] identified impaired subscapularis integrity at the time of surgery as the most important risk factor for dislocations in shoulders where reconstruction was impossible due to insufficient proximal humerus bone stock, no higher risk was seen in patients with cuff tear arthropathy as aetiology. Unfavourable biomechanical properties after RSA, as shown in this study, with a decreased moment arm in conjunction with the decreased muscle tension might impede better results, no matter if the subscapularis is reconstructed or not. On the other hand, its integrity might have been irreversibly impaired pre-operatively or secondary to the surgical approach.

Differences of the origin-to-insertion distances were most pronounced for the cranial segments in the 15 and 30 degrees abduction positions for both muscles. With increasing abduction this difference decreases and for some segments and positions no significant difference can be seen. We assume that with implantation of the RSA and distalisation of the humerus an increased distance of the tendon insertions to the rotational center arises. This results in a more eccentric motion of these landmarks and might explains the decrease of the origin-to-insertion distance with increasing abduction.

In both muscles some segments had positive abduction moment arms preoperatively, which in healthy shoulders is essential for their function as dynamic stabilisers of the shoulder joint. The loss of this function will lead to a smaller joint compression force and as a result increase subluxation forces[28]. These increased forces might abet glenoid loosening and instability. No beneficial effect can be seen for the increased postoperative adduction moment arms as adduction is usually not impaired in patients with cuff arthropathy, neither pre- nor postoperatively.

Scapular notching is one major complication in reverse shoulder arthroplasty[29]. Mechanical impingement as well as secondary bone erosion due to polyethylene wear is believed to contribute to this phenomenon. In our study, inferior impingement between the humeral component and the scapular neck was only observed in the zero degree reference position, which, however, is not the neutral thoraco-humeral position, but rather an adduction position which is not of high clinical relevance. Even though scapular notching was not the specific focus of this study, these findings are in agreement with the observations of other authors[30] on this subject.

## Conclusion

In conclusion, this study is the first to analyse the moment arms and the change in the distance between muscle insertion sites of the remaining rotator cuff after RSA. During glenohumeral abduction, significant changes were seen in both, the teres minor and the subscapularis moment arms. These changes may contribute to the clinically observed functional deficits.

## Competing interests

The authors declare that they have no competing interests.

## Authors' contributions

SH, CK, and SG contributed to conception and design of the study, acquisition of data, analysis and interpretation of data, and drafting the manuscript. CK and MH derived the mathematical model. SG and CP helped to draft the manuscript and supervised the whole study. All authors read and approved the final manuscript.
